# Evaluation of Paired-End Sequencing Strategies for Detection of Genome Rearrangements in Cancer

**DOI:** 10.1371/journal.pcbi.1000051

**Published:** 2008-04-25

**Authors:** Ali Bashir, Stanislav Volik, Colin Collins, Vineet Bafna, Benjamin J. Raphael

**Affiliations:** 1 Bioinformatics Graduate Program, University of California San Diego, San Diego, California, United States of America; 2 Comprehensive Cancer Center, University of California San Francisco, San Francisco, California, United States of America; 3 Department of Computer Science and Engineering, University of California San Diego, San Diego, California, United States of America; 4 Department of Computer Science and Center for Computational Molecular Biology, Brown University, Providence, Rhode Island, United States of America; European Bioinformatics Institute, United Kingdom

## Abstract

Paired-end sequencing is emerging as a key technique for assessing genome rearrangements and structural variation on a genome-wide scale. This technique is particularly useful for detecting copy-neutral rearrangements, such as inversions and translocations, which are common in cancer and can produce novel fusion genes. We address the question of how much sequencing is required to detect rearrangement breakpoints and to localize them precisely using both theoretical models and simulation. We derive a formula for the probability that a fusion gene exists in a cancer genome given a collection of paired-end sequences from this genome. We use this formula to compute fusion gene probabilities in several breast cancer samples, and we find that we are able to accurately predict fusion genes in these samples with a relatively small number of fragments of large size. We further demonstrate how the ability to detect fusion genes depends on the distribution of gene lengths, and we evaluate how different parameters of a sequencing strategy impact breakpoint detection, breakpoint localization, and fusion gene detection, even in the presence of errors that suggest false rearrangements. These results will be useful in calibrating future cancer sequencing efforts, particularly large-scale studies of many cancer genomes that are enabled by next-generation sequencing technologies.

## Author Summary

Cancer is driven by genomic mutations that can range from single nucleotide changes to chromosomal aberrations that rearrange large pieces of DNA. Often, these chromosomal aberrations disrupt a gene sequence, and even fuse the sequences of two genes, producing a “fusion gene.” Fusion genes have been identified as key participants in the development of several types of cancer. Using genome-sequencing technology it is now possible to identify chromosomal aberrations genome-wide and at high resolution. In this paper, we address the question of how much sequencing is required to detect a chromosomal aberration and to determine the location of the aberration precisely enough to identify if a fusion gene is created by this aberration. We derive a mathematical formula that accurately predicts a number of fusion genes in a breast cancer sequencing study. We also demonstrate how the ability to detect chromosomal aberrations and fusion genes depends on both the size of the fusion gene and the parameters of the genome sequencing strategy that is used. These results will be useful in calibrating future cancer sequencing efforts, especially those using next-generation sequencing technologies.

## Introduction

Cancer is a disease driven by selection for somatic mutations. These mutations range from single nucleotide changes to large-scale chromosomal aberrations such as deletion, duplications, inversions and translocations. While many such mutations have been cataloged in cancer cells via cytogenetics, gene resequencing, and array-based techniques (i.e. comparative genomic hybridization) there is now great interest in using genome sequencing to provide a comprehensive understanding of mutations in cancer genomes. The Cancer Genome Atlas (http://cancergenome.nih.gov/index.asp) is one such sequencing initiative that focuses sequencing efforts in the pilot phase on point mutations in coding regions. This approach largely ignores copy *neutral* genome rearrangements including translocations and inversions. Such rearrangements can create novel fusion genes, as observed in leukemias, lymphomas, and sarcomas [1]–[3]. The canonical example of a fusion gene is BCR-ABL, which results from a characteristic translocation (termed the “Philadelphia chromosome”) in many patients with chronic myelogenous leukemia (CML) [3]. The advent of Gleevec, a drug targeted to the BCR-ABL fusion gene, has proven successful in treatment of CML patients [4], invigorating the search for other fusion genes that might provide tumor-specific biomarkers or drug targets.

Until recently, it is was generally believed that recurrent translocations and their resulting fusion genes occurred only in hematological disorders and sarcomas, with few suggesting that such recurrent events were prevalent across all tumor types including solid tumors [5],[6]. This view has been challenged by the discovery of a fusion between the TMPRSS2 gene and several members of the ERG protein family in prostate cancer [7] and the EML4-ALK fusion in lung cancer [8].

**Figure 1:**
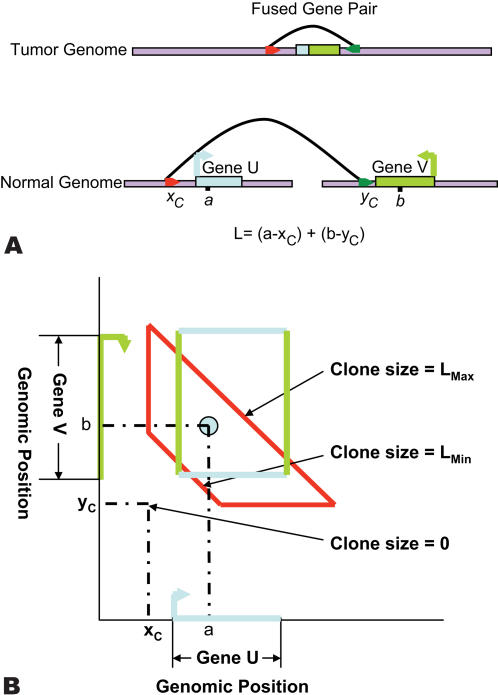
Schematic of breakpoint calculation. (A) The endpoints of a clone *C* from the cancer genome map to locations *x_C_* and *y_C_* (joined by an arc) on the reference genome that are inconsistent with *C* being a contiguous piece of the reference genome. This configuration indicates the presence of a breakpoint (*a*,*b*) that fuses at ζ in the cancer genome. (B) The coordinates (*a*,*b*) of the breakpoint are unknown but lie within the trapezoid described by Equation 1. The observed length of the clone is given by *L_C_* = (*a*−*x_C_*)+(*b*−*y_C_*). The rectangle *U*×*V* describes the breakpoints that lead to a fusion between genes *U* and *V*.

These studies raise the question of what other recurrent rearrangements remain to be discovered. One strategy for genome-wide high-resolution identification of fusion genes and other large scale rearrangements is paired-end sequencing of clones, or other fragments of genomic DNA, from tumor samples. The resulting end-sequence pairs, or *paired reads*, are mapped back to the reference human genome sequence. If the mapped locations of the ends of a clone are “invalid” (i.e. have abnormal distance or orientation) then a genomic rearrangement is suggested (See Figure 1 and Methods). This strategy was initially described in the End Sequence Profiling approach [9] and later used to assess genetic structural variation [9],[10]. An innovative approach utilizing SAGE-like sequencing of concatenated short paired-end tags successfully identified fusion transcripts in cDNA libraries [11]. Present and forthcoming next-generation DNA sequencers hold promise for extremely high-throughput sequencing of paired-end reads. For example, the Illumina Genome Analyzer will soon be able to produce millions of paired reads of approximately 30 bp from fragments of length 500–1000 bp [12], while the SOLiD system from Applied Biosystems promises 25 bp reads from each end of size selected DNA fragments of many sizes [13]. Similar strategies coupling the generation of paired-end tags with 454 sequencing have also been described [14],[15].

Whole genome paired-end sequencing approaches allow for a genome-wide survey of all potential fusion genes and other rearrangements in a tumor. This approach holds several advantages over transcript or protein profiling in cancer studies. First, discovery of fusion genes using mRNA expression [7], cDNA sequencing, or mass spectrometry [16] depends on the fusion genes being transcribed under the specific cellular conditions present in the sample at the time of the assay. These conditions might be different than those experienced by the cells during tumor development. Second, measurement of fusions at the DNA sequence level focuses on gene fusions due to genomic rearrangements and thus is less impeded by splicing artifacts or *trans* splicing [17]. Finally, genome sequencing can identify more subtle regulatory fusions that result when the promoter of one gene is fused to the coding region of another gene, as in the case with with the c-Myc oncogene fusion with the immunoglobin gene promoter in Burkitt's lymphoma [18].

In this paper, we address a number of theoretical and practical considerations for assessing cancer genome organization using paired-end sequencing approaches. We are largely concerned with detecting a rearrangement breakpoint, where a pair of non-adjacent coordinates in the reference genome is adjacent (i.e. fused) in the cancer genome. In particular, we extend this idea of a breakpoint to examine the ability to detect fusion genes. Specifically, if a clone with end sequences mapping to distant locations identifies a rearrangement in the cancer genome, does this rearrangement lead to formation of a fusion gene? Obviously, sequencing the clone will answer this question, but this requires additional effort/cost and may be problematic; e.g. most next-generation sequencing technologies do not “archive” the genome in a clone library for later analysis (for the sake of simplicity we will use the term “clone”to refer to any contiguous fragment that is sequenced from both ends). We derive a formula for the probability of fusion between a pair of genomic regions (e.g. genes) given the set of all mapped clones and the empirical distribution of clone lengths. These probabilities are useful for prioritizing follow-up experiments to validate fusion genes. In a test experiment on the MCF7 breast cancer cell-line, 3,201 pairs of genes were found near clones with aberrantly mapping end-sequences. However, our analysis revealed only 18 pairs of genes with a high probability (>0.5) of fusion, of which six were tested and five experimentally confirmed (Table 1).

**Table 1:** Fusion probability predictions and sequencing results for clusters in breast cancer.

Start Gene	End Gene	Fusion Probability	Cluster Size	Sequencing Supporting Fusion	Cell Line/Primary Tumor
ASTN2	PTPRG	1	2	Yes†	MCF7
BCAS4	BCAS3	1	20	Yes†	MCF7
KCND3	PPM1E	0.99	12	Yes	MCF7
NTNG1	BCAS1	0.99	6	Yes	MCF7
BCAS3	ATXN7	0.83	8	Yes†	MCF7
ZFP64	PHACTR3	0.6322	2	No	BT474
CT012_HUMAN	UBE2G2	0.0880	1	No	Breast
VAPB	ZNFN1A3	0.0842*	3	Yes	BT474
BMP7	EYA2	0.0324	4	No†	MCF7
KCNH7	TDGF1	0.0215	1	No	Breast
SULF2	TBX4	0.00656	2	No	MCF7
NACAL	NCOA3	0.0057	2	No	MCF7
MRPL45	TBC1D3C	0.0005	1	No	BT474
U1	NP_060028.2	0.0005	1	No	Breast
RBBP9	ITGB2	0.0005	1	No	Breast
Y	SYNPR	<0.0001	4	No	MCF7
PRR11	TMEM49	<0.0001	9	No	MCF7
BMP7	Q96TB	<0.0001	3	No	MCF7

The gene order shown indicates “start” and “end” positions with respect to the direction of transcription. Note that *VAPB/ZNFNA13* has low probability of fusion, but there are many pairs of genes with low probability of fusion in this region. The probability that any *one* of these gene pairs fuse is >.30. All clones in a cluster are non-redundant (the same clones do not reappear multiple times in a cluster). Additional clones have been sequenced [22], but these did not overlap *any* predicted fusion genes – these sequenced clones were also found not to contain fusion genes.

**†:** A single clone contained more than two chromosomal segments, i.e. the clone is not a simple fusion of two genomic loci.

The advent of high throughput sequencing strategies raises important experimental design questions in using these technologies to understand cancer genome organization. Obviously, sequencing more clones improves the probability of detecting fusion genes and breakpoints. However, even with the latest sequencing technologies, it would be neither practical nor cost effective to shotgun sequence and assemble the genomes of thousands of tumor samples. Thus, it is important to maximize the probability of detecting fusion genes with the least amount of sequencing. This probability depends on multiple factors including the number and length of end-sequenced clones, the length of genes that are fused, and possible errors in breakpoint localization. Here, we derive (theoretically and empirically) several formulae that elucidate the trade-offs in experimental design of both current and next-generation sequencing technologies. Our probability calculations and simulations demonstrate that even with current paired-end technology we can obtain an extremely high probability of breakpoint detection with a very low number of reads. For example, more than 90% of all breakpoints can be detected with paired-end sequencing of less than 100,000 clones (Table 2). Additionally, next-generation sequencers can potentially detect rearrangements with a greater than 99% probability and localize the breakpoints of these rearrangements to intervals of less than 300 bp in a single run of the machine (Table 2).

**Table 2:** Breakpoint detection and localization for different sequencing strategies.

Clone Length(L)	Paired Reads (N)	Clone Coverage (c)	*E* (|Θ_ζ_|)	*P*_ζ_	*E* (|Θ_ζ*_|)	*P*_ζ*_
1 kb	40×10^6^	13.3×	295	>.99	289	.99
1 kb	1×10^6^	.33×	972	.15	658	.012
2 kb	20×10^6^	13.3×	593	>.99	581	.99
2 kb	1×10^6^	.66×	1889	.28	1296	.044
10 kb	5×10^6^	16.7×	2393	>.99	2378	>.99
10 kb	1×10^6^	3.3×	7342	.81	5657	.50
40 kb	2×10^6^	26.7×	5998	>.99	5997	>.99
40 kb	.1×10^6^	1.33×	35587	.49	25124	.14
150 kb	.5×10^6^	25×	23997	>.99	76807	.71
150 kb	.1×10^6^	5×	93169	.92	72022	.80
150 kb	.012×10^6^	.6×	142510	.26	97457	0.037

The probability *P*_ζ_ of detecting a fusion point and the expected length *E*(|Θ_ζ_|) of a breakpoint region under various clone lengths (*L*) and number of end-sequenced clones (*N*). The values of *N* and *L* are chosen to reflect current or proposed sequencing platforms, with the last value for the 150 kb clones representing our current status on the MCF7 cell line. *P*_ζ*_ and *E*(|Θ_ζ*_|) correspond to the probability for, and expected size of, a breakpoint region in the case when *two* clones are required to span ζ. The small clone lengths (1 kb, 2 kb) and large number of reads represent what one might achieve in a single run with new technologies (under perfect mapping of end sequences). For a comparison of *E*(|Θ_ζ_|) and *N* for a fixed *P*_ζ_ = .99 over a continuous range of clone lengths, see Figure S6.

## Results

### Computing the Probability of Fusion Genes

Given a set of clones from a cancer genome, we want to compute the probability that these clones identify a fusion gene in the cancer genome, i.e. a fusion of two different genes from the reference genome. We consider the cancer genome as a rearranged version of the reference human genome and assume that there exists a mapping between coordinates of the two genomes. The reference genome is described by a single interval of length *G*; i.e. we concatenate multiple chromosomes into a single coordinate system. We define a *breakpoint* (*a*,*b*) as a pair of non-adjacent coordinates *a* and *b* in the reference genome that are adjacent in the cancer genome. Correspondingly, we define the *fusion point* as the coordinate ζ in the cancer genome such that the point *a* maps to ζ and the point *b* maps to ζ+1. Note that in the genome rearrangement literature, a fusion point is also called a breakpoint [19]. Consider a clone *C* containing ζ. If the breakpoints *a* and *b* are far apart (e.g. on different chromosomes) then the endpoints of *C* will map to two locations, *x_C_* and *y_C_*, on the reference genome that are inconsistent with *C* being a contiguous fragment of the reference genome (Figure 1A). In this case, we say that (*x_C_*,*y_C_*) is an *invalid pair* [20]. Observing an invalid pair (*x_C_*,*y_C_*) does not identify the breakpoint (*a*,*b*) exactly. However, if we know that the length of the clone *C* lies within the range [*L*_min_,*L*_max_], and we assume that: (i) only a *single* breakpoint is contained in a clone; and (ii) *a*>*x_C_* and *b*>*y_C_* (without loss of generality: See Methods); then breakpoint (*a*,*b*) that are consistent with (*x_C_*,*y_C_*) must satisfy
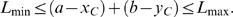
(1)If we plot an invalid pair (*x_C_*,*y_C_*) as a point in the two dimensional space *G*×*G* then the breakpoints (*a*,*b*) satisfying the above equation define a trapezoid (or triangle when *L*_min_ = 0) (Figure 1).

**Figure 2:**
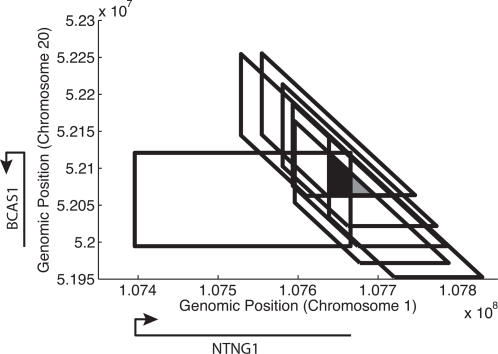
Prediction of a fusion between the NTNG1 and BCAS1 genes. The rectangle indicates the possible locations of a breakpoint on chromosomes 1 and 20 that would result in a fusion between NTNG1 and BCAS1. Each trapezoid indicates possible locations for a breakpoint consistent with an invalid pair. Assuming that all clones contain the same breakpoint, this breakpoint must lie in the intersection of the trapezoids (shaded region). Approximately 69% of this shaded region intersects (darkly shaded region) the fusion gene rectangle, giving a probability of fusion of approximately 0.69. The empirical distribution of clone lengths reveals that not all clone lengths are equally likely (e.g. extremely long or short clones are rare). Using this additional information, our improved estimate for the probability of fusion is >0.99.

If multiple clones contain the same fusion point ζ, then the corresponding breakpoint (*a*,*b*) lies within the intersection *I* of the trapezoids corresponding to the clones. Conversely, we will assume that if the trapezoids defined by several invalid pairs intersect, then they share a common breakpoint. We call a set of clones whose trapezoids have non-empty intersection a *cluster*. Figure 2 displays a cluster of six clones from the MCF7 breast cancer cell line. As the number of clones that are end-sequenced increases, more clones will contain the same fusion point and more clusters will be formed. Thus, the area of *I* will decrease, and therefore the uncertainty in the location of the fusion point decreases.

Now, each gene in the reference genome defines an interval *U* = [*s*,*t*] where *s* is the 5′ transcription start site and *t* is the 3′ transcription termination site. Consider two genes with intervals *U* and *V*. The two genes are fused if there exists a breakpoint (*u*,*v*) that lies in *U*×*V*. This breakpoint is detected if (*u*,*v*) lies in *I*. Thus, an approximate probability for the existence of a fusion gene is the fraction of *I* that lies within the rectangle *U*×*V*. We obtain a better estimate of the probability of fusion by considering the *empirical* distribution of clone lengths. The exact probability of the gene fusion is given by the probability mass that lies within the intersection of *I* and the rectangle *U*×*V* defined by the pair of genes. An efficient algorithm for computing these probabilities is given in Methods.

### Fusion Gene Predictions in Breast Cancer

We made predictions of fusion genes for the MCF7, BT474, and SKBR3 breast cancer cell lines as well as two primary tumors using data from end sequence profiling of these samples [21],[22]. Approximately, 71 Mb of end-sequence was obtained from these 5 samples, ∼29 Mb (corresponding to .47 clonal coverage) coming from the MCF7 cell line. Across all samples, a total of 1,141 invalid pairs were obtained. These formed 919 clusters, 95 of which contained more than one clone.

**Figure 3:**
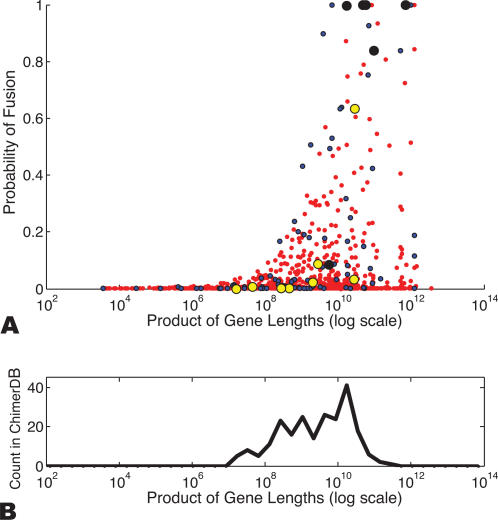
Fusion genes and gene lengths. (A) Probability of fusion vs. the product of gene lengths involved in the fusion indicates higher fusion probabilities for pairs of larger genes. Larger circles indicate gene pairs experimentally validated by further sequencing. A “Positive Result” indicates a predicted fusion for which sequencing results supported a fusion gene. A “Negative Result” indicates a predicted fusion for which sequencing results did not support a fusion gene. (B) The number of fusion genes in chimerDB [23] plotted as a function of the product of gene lengths in the fusion.

We applied our method of computing fusion gene probability to each of these samples, using the distribution of clone lengths in each library for these calculations. Figure S1 shows this empirical distribution for the MCF7 library. Table 1 shows the results of our predictions for fully sequenced BACs across multiple breast cancer cell lines and primary tumors, sorted according to fusion probability. We have successfully validated a number of these highest ranked predictions by sequencing the *entire* clone and identifying the exact location of the breakpoint and point of gene fusion (See Methods). Sequencing also showed that certain clones contain *multiple* rearrangement breakpoints with more than two contiguous segments of the reference genome present in a single clone (Table 1). In these cases, we ensure that the breakpoint associated to each gene in the fusion disrupts the corresponding gene. Such multiple rearranged regions have been observed to still form fusion transcripts as in the case of BCAS4/BCAS3 [11],[23]. Figure 2 illustrates the computation of fusion probability for one high-scoring prediction (NTNG1/BCAS1). The strong correspondence between fusion probability prediction and subsequent validation of the breakpoints by sequencing in Table 1 illustrates the predicative power of our method. Table 1 also indicates the power of the technique in predicting clones that do not have fusion genes. Only one clone with fusion probability below 50% contained a fusion gene (*VAPB/ZNFN1A3*). The data suggests a strong correlation between gene rectangle size (the product of gene lengths) and probability of gene fusion. Larger fusion genes tend to have higher fusion probabilities and greater likelihood of being validated (Figure 3). A similar trend is observed in chimerDB, a database of fusion genes in cancer derived from mRNA, EST, literature and database searches [24].

### Detection and Localization of Genome Rearrangements

We now consider the problem of how much sequencing is required to detect a genome rearrangement and to localize the breakpoint of a rearrangement. Consider an idealized model in which *R* clones, each of fixed length *L* are picked uniformly at random from a cancer genome of length *G* (where *G* will equal the diploid genome size, ∼6×10^6^ bp) and end-sequenced. These end sequences are mapped to the reference genome and the fraction *f* of clones with uniquely mapped ends yields *N* = *fR* clones that can be used to identify rearrangements. The fraction *f* of clones uniquely mapped varies significantly among different sequencing technologies. In an ESP study, with paired-end Sanger sequencing of BACs, 90% of reads were mappable with 58% uniquely mapped [22]. A recent study that used 454 sequencing to identify structural variants in the human genome reported 63% mapping of sequences with recognizable linker sequences, and 41% of all reads mapped [15]. Note that the 454 reads are of significantly longer (average 109 bp) compared to other next generation sequencing technologies (average 20–30 bp) [12],[13],[15] and thus even lower mapping efficiencies are expected for these shorter reads.

**Figure 4:**
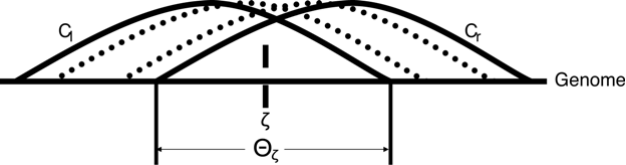
Schematic of a breakpoint region. A fusion point ζ on the cancer genome contained in multiple clones. The leftmost and rightmost clones determine the breakpoint region Θ_ζ_ in which the fusion point can occur.

A fusion point, ζ, on the cancer genome is detected if a uniquely mapped clone contains it (Figure 4). Using the Clarke-Carbon formula [25],[26] (See Methods), the probability *P*_ζ_ of detection of ζ is given by
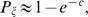
(2)where *c* = *NL*/*G* is the *clonal coverage*. If only a single clone contains a fusion point, then the fusion point is localized to within *L* bp. If multiple clones contain a fusion point, then the fusion point is localized more precisely. We define the breakpoint region, Θ_ζ_, as the interval determined by the intersection of all clones that contain ζ. Thus, |Θ_ζ_| defines the localization of ζ, or the uncertainty in mapping ζ. Since localizing a fusion point to within *L*, requires only a single clone containing ζ, we find (see Methods) that
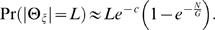
(3)Furthermore, we find that for *s*<*L*,
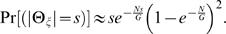
(4)


These equations allow us to estimate the expected length of Θ_ζ_, *conditioned* on ζ being covered (otherwise, Θ_ζ_ is not defined) as
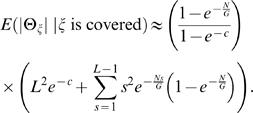
(5)See Methods section for full derivation and closed form solution (Equation 24). We evaluated the error in this approximation by simulation (See Text S1 for descriptions of all simulations). Figure S2 shows that Equation 5 very closely models the average observed |Θ_ζ_|. The relative error between the average observed length of the breakpoint region and Equation 5 was 0.02.

**Figure 5:**
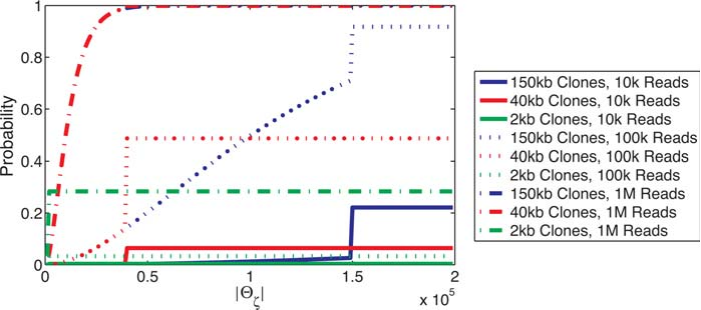
Probability of localizing a fusion point to an interval of a given length. A fusion point ζ is localized to length *s* if the corresponding breakpoint point region Θ_ζ_ has length *s* or less. When *s* exceeds the clone length *L*, only a single clone is required to achieve this localization and consequently the probability of localization is the probability that at least one clone contains the fusion point. In the case of 1 M paired reads the 40 kb and 150 kb curves are nearly indistinguishable. Note that each curve is obtained using a fixed clone length, and that the use of a distribution of clone lengths would create a less abrupt transition.

We also assessed the effect of different clone lengths, *L*, and number of clones, *N*, on the expected length of the breakpoint region, *E*(|Θ_ζ_|), around a specific fusion point, ζ. Figure 5 shows that as the number of reads, *N*, increases, the uncertainty in localization (|Θ_ζ_|) decreases. Interestingly, note that the 40 kb clones are most advantageous when localization |Θ_ζ_| = 40 kb is desired. A similar effect was observed for the 150 kb and 2 kb clones. Thus, there is a direct correlation between the clone length and the ability to *localize* a fusion point to a given sized interval, implying that the choice of clone lengths impacts the ability to detect fusions of a specific size.

### Comparison of Sequencing Strategies

Formulas 2 and 5 provide a framework for examining a variety of choices of sequencing parameters *L*, *N*, and *c*. Table 2 and Figures S3, S4, and S5 demonstrate the effect of using different clone lengths and varying numbers of paired reads on the ability to detect and localize a fusion point. Table 2 also indicates the effect of such parameters on the ability to detect and localize *clusters* of invalid pairs, as defined by Formulas 25 and 26. One can see that a distinct trade-off exists between detection, in which larger clones hold a distinct advantage, and localization, in which case smaller clones are advantageous. Longer clones (e.g. BACs of 150 kb) are more pragmatic for sequencing projects using a smaller number of paired reads, but the advent of low cost, highly parallel sequencing of small clones could soon yield extremely high probability of detection (high *P*_ζ_) and extremely high resolution of fusion points (small |Θ_ζ_|).

### Lengths of Fusion Genes

**Figure 6:**
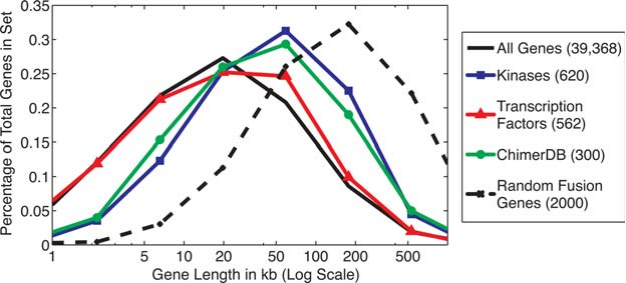
Distribution of gene sizes for different groups of genes. All genes: The “known genes” track in the UCSC Genome Browser [27]. Kinases: Selected from the KinBase database [36]. Transcription factors: Selected from the AmiGO database according to the GO term “transcription factor activity” [37]. ChimerDB: Fusion genes in cancer extracted from the chimerDB database [23]. Random Fusion Genes: A set of 2000 genes involved in 1000 random fusion events. Random Fusion events were formed by inducing random breakpoints, and selecting such events if they formed a fusion gene. Note that the gene sizes are on a log scale, and the number of genes from each set used to derive each distribution is shown in the legend.

Since our simulations revealed that the choice of sequencing parameters affects the ability to localize breakpoint regions to intervals of different lengths (Figure 5), we further explored what lengths might be advantageous for identification of fusion genes. There is considerable variation in sizes of human genes (Figure 6). When considering all known transcripts [27], the median gene size is approximately 20 kb and the mean is approximately 40 kb. However, examination of chimerDB shows a clear bias towards larger genes, with a median gene size of 40 kb and a mean gene size of 90 kb. It is tempting to speculate on the reasons for this bias. One possibility is ascertainment bias, as larger fusion genes would be easier to identify via cytogenetic techniques which to date have been the technique used to identify most fusion genes. Additionally, random breakage of the genome would favor fusions involving larger genes, as the probability of a breakpoint disrupting a large gene would be greater than for a small gene. We examined the length distribution of random fusion genes by simulation. We selected random breakpoints in the genome, and if a breakpoint formed a fusion gene we recorded the length of the resulting fusion gene (Figure 6). It is interesting to note that these random fusion events resulted in much larger genes than observed in the normal genome *or* chimerDB (median and mean gene sizes of 155 kb and 284 kb, respectively). Though further investigations are needed, one possible explanation is that known fusion genes have a biased size distribution because they are selected for functional reasons. We also examined the distribution of transcription factor genes and kinase genes, both of which are members of multiple fusion genes (Figure 6). Interestingly, the size distribution of kinases is closer to the chimerDB distribution, while the size distribution of transcription factors is closer to the size distribution of all known genes.

**Figure 7:**
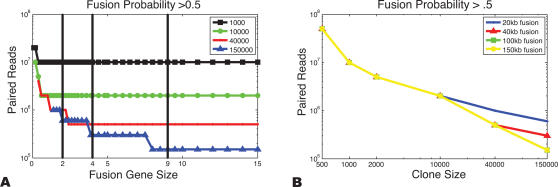
The number of paired reads necessary to detect fusion genes. (A) The number of paired reads necessary to detect fusion genes with fusion probability greater than 0.5 as a function of gene size for different clone lengths. The vertical lines indicate median (20 kb) and mean (40 kb) sizes for all known genes as well as the median (40 kb) and mean (90 kb) sizes for chimerDB genes. (B) The number of paired reads necessary to detect fusion genes with fusion probability greater than 0.5 as a function of clone length for different fusion genes sizes (log scale in both axes). Each point in these plots is the average over 100 different fusion genes and and 100 different simulations of clone sets from the genome. Thus, each data point represents the average value of 10^4^ simulations. In each simulation, a pair of genes was chosen such that area of the resulting gene rectangle (*U*×*V*) was equal to the square of the indicated fusion gene size. A breakpoint was selected for the gene pair uniformly in the rectangle *U*×*V*).

The variation in gene sizes for different classes of genes (Figure 6) suggests that one consider a wide range of gene sizes when assessing our ability to detect fusion genes. Figure 7 shows the number of clones, for different lengths, that are required to achieve a fusion probability greater than 0.5 for a random gene pair of fixed size. Note that the breakpoint could exist at *any* position within either gene. Smaller clone sizes clearly hold a distinct advantage in fusion probability for equal *clonal* coverage while large clones perform better for a fixed number of paired reads (Figure 7A). This is not surprising, as a significantly higher number of *paired reads* is required to achieve the same coverage with smaller clones. In particular, 75 times more paired reads from 2 kb clones are needed to obtain the same clonal coverage as 150 kb clones.

There is also a relationship between the size of a fusion gene and the probability of detecting the fusion (Figure 7B). Since larger clones create larger trapezoids (Figure 1) the use of larger clones increases the probability that the trapezoid defined by the clone intersects the rectangle defined by the two genes, thus producing a higher probability of detection of a breakpoint. However, this effect is counteracted by the fact that larger clones also yield larger *breakpoint regions*, leading to lower fusion probabilities since only a small fraction of a larger trapezoid typically overlaps the gene rectangle. The optimal clone length for fusion gene identification is directly related to the length of fusion genes. Thus, the length of fusion genes that one wants to detect with high probability is an important parameter in choosing a sequencing strategy. For example, if a fusion gene is 40 kb in length, the average fusion probability is significantly greater when using the same number of 40 kb clones compared to 2 kb or 10 kb clones, because of the greater genomic coverage provided by the larger clones. However, in this scenario 40 kb clones also perform nearly as well as 150 kb clones (Figure 7B), because the 40 kb clones have better breakpoint localization (Figure 5). If the fusion gene size is increased to 150 kb, then 150 kb clones are superior since the poorer breakpoint localization has limited effect on prediction of a large fusion gene. One additional consideration is that larger clones (e.g.150 kb) consistently show lower variance in fusion probabilities (Figure S7) due to their higher probability of detecting a fusion. This makes larger clones more reliable when performing studies across multiple tumor samples, especially when the number of paired reads available for its sample is limited.

### Effects of Errors

There are numerous sources of error in paired-end sequencing strategies for rearrangement identification including experimental artifacts, genome assembly errors or mis-mapping of end sequences. These errors can lead to incorrect predictions of fusion genes, or false positives. A major source of experimental artifacts in current sequencing approaches is chimeric clones that are produced when two non-contiguous regions of DNA are joined together during the cloning procedure. Approximately 1–2% of clones in modern BAC libraries are chimeric [21], and rates for other vectors are roughly similar [15]. The type and rate of experimental artifacts for new genome amplification and sequencing strategies is still an open question.

**Figure 8:**
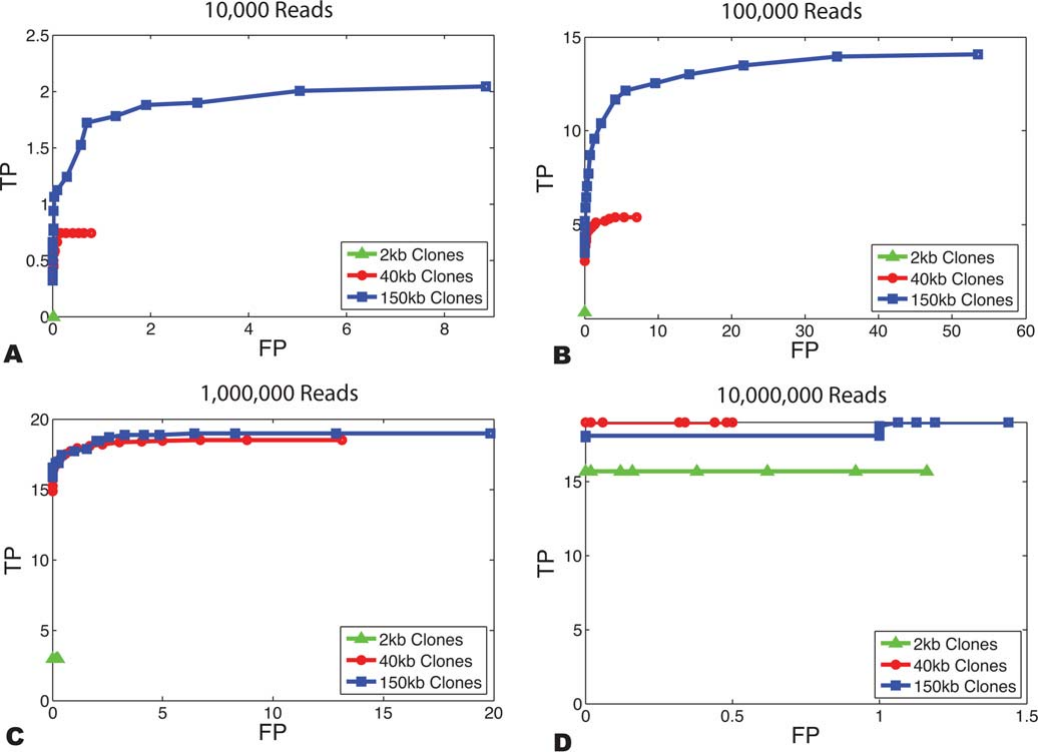
Sensitivity and specificity of fusion gene predictions. (A) Number of false positive (FP) and true positive (TP) fusion gene predictions for a simulated genome with 100 translocations and 10,000 paired reads. Each curve represents the average of 50 simulations with clones of a fixed length (2 kb, 40 kb, 150 kb clones). The minimum fusion probability threshold for indicating that a fusion gene was predicted was decreased from >.95 (leftmost point) to >0 (rightmost point) in increments 0.05 and the number of true and false predictions was determined. For all figures 19 true fusion genes were present in the rearranged genome. These 19 events were not selected for but rather they resulted from random rearrangement of the genome. (B) 100,000 paired reads. (C) 1,000,000 paired reads. (D) 10,000,000 paired reads.

In order to assess the rate of false positive predictions of fusion genes in the presence of errors, we simulated 100 random genome rearrangements with 1% of the paired-end sequences arising from chimeric clones. For several clone lengths, we recorded the number of fusion genes correctly identified (true positives) and the number of incorrect fusion gene predictions (false positives) as the minimum fusion gene probability required for identification was increased (Figure 8). For small numbers of paired reads, the largest clones (150 kb) yield the largest number of true positives (Figure 8A and 8B), while with a large number of paired reads, smaller clones (40 kb) are better (Figure 8C). Extremely large numbers of paired reads are required before very small clones (2 kb) become effective (Figure 8D). On the other hand, these small clones show almost no false positives at reasonable probability thresholds, and show little (if any) increase in true positives if the probability threshold is reduced (Figure 8D).

**Figure 9:**
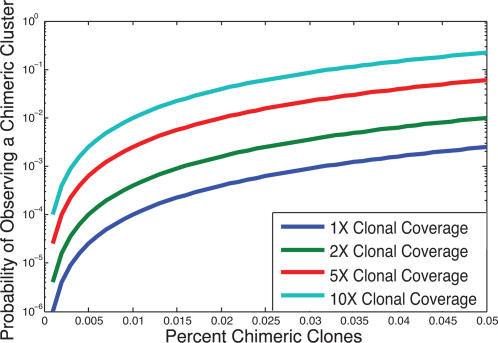
Probability of observing at least one chimeric cluster vs. the percent of chimeric clones. These probabilities were computed using Equation 27, with clone length *L* = 150 kb and confirmed by simulation. Other clone lengths yield virtually identical probabilities at the same *clonal coverage*. Note: the y-axis is log scaled.

Finally, we examined the effect of chimeric clones on our ability to identify breakpoints from invalid *clusters*. Obviously, when only a single isolated invalid pair exists we cannot determine whether it arose from a chimeric clone or from a true rearrangement. However, a *cluster* of invalid pairs is highly unlikely to arise from chimeric clones [20]. Figure 9 shows that in most cases, no clusters of chimeric clones are observed. Even under high coverage (10× clonal coverage) and a very high percentage of chimeric clones (5% of all paired reads) 80% of the time no chimeric clusters were observed. This result demonstrates that clusters of two or more invalid pairs are very likely to indicate true rearrangement events. When comparing a fixed number of chimeric clones over clones of varying lengths, the probability of observing a chimeric cluster is much lower for smaller clones (Figure S8).

## Discussion

We provided a computational framework to evaluate paired-end sequencing strategies for detection of genome rearrangements in cancer. Our probability calculations and simulations show that current paired-end technology can obtain an extremely high probability of breakpoint detection with a very low number of reads. For example, more than 90% of all breakpoints can be detected with paired-end sequencing of less than 100,000 clones (Table 2). Additionally, next-generation sequencers can potentially detect rearrangements with greater than 99% probability and localize the breakpoints of these rearrangements to intervals less than 300 bp in a single run of the machine (Table 2). If only a fraction (e.g. 50%) of the reads map uniquely, similar detection levels are achievable by simply doubling the amount of sequencing.

We derived formulae that provide estimates of the probability of detecting rearrangement breakpoints and localizing them precisely. For a genome of length *G* with *N* mapped paired reads from clones of length *L*, the detection probability is a function of the of clonal coverage (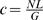). Thus, increasing *L* means that fewer clones are needed to maintain the same probability of detecting a fusion. On the other hand, breakpoint localization depends independently on “clone” length *L*, number of mapped reads *N*, and genome size *G*. Traditionally, clone length *L* was dictated by efficiency considerations with available cloning vectors (e.g. plasmids≈2 kb, fosmids≈40 kb, and BACs≈150 kb). However, new sequencing technologies permit paired-end sequencing from a larger range of “clone” lengths.

The natural question for the practitioner is: what sequencing strategy maximizes information about rearrangements in the cancer genome for minimum cost? Three considerations preclude a definitive answer to the question. First, the goal of “maximizing information about rearrangements” in cancer genomes requires further specification. Second, the parameters of a sequencing strategy cannot be set arbitrarily, but are restricted by the chosen technology. Third, the complexity of cancer genomes at the sequence level – including the number and type of rearrangements and the sequence characteristics of rearrangement breakpoints – is currently unknown We discuss each of these issues below and then conclude by describing further extensions of our methodology.

### Defining the Genomic Features of Interest

When studying genome rearrangements by paired-end sequencing approaches, there are two interrelated goals that affect the choice of sequencing strategy. First, one might be interested in detecting as many rearrangement breakpoints as possible with the minimum amount of sequencing. In this case, the goal is to maximize the clonal coverage *c* with the fewest number of paired reads. It follows immediately from the breakpoint detection probability (Equation 2) that for a fixed number of paired reads, larger clones give higher probabilities of detection than smaller clones. On the other hand, one might be interested in precise localization of breakpoint regions. In this case, smaller clones provide better localization *when* the breakpoint is detected (Figure 8, Figure S5).

Better localization of breakpoints is desirable if one wants to determine with certainty that a gene is fused or disrupted by a genome rearrangement. Our results showed the correlation between clone length and the probability of localizing breakpoints to an interval of a specific length. Figure 5 shows that with a fixed number of paired reads, the optimal choice of clone length depends on the desired interval of localization. Figure 7B shows that these results readily translate to the probability of detecting fusion genes of a given size. If paired-end sequences could be obtained for any clone length, then the choice of optimal clone length depends on the length of fusion genes that the researcher wants to have the greatest ability to localize. This in turn might depend on a prior belief about the model of rearrangement in cancer. For example, if one wants to be able to localize fusion genes whose length is approximately the length of an average human gene (40 kb), then the optimal clone length is 40 kb. However, under the hypothesis that the breaks in the genome that lead to fusion genes are distributed uniformly on the genome, larger fusion genes would be expected and thus larger clones would be optimal.

Better localization is also desirable when one wants to validate a breakpoint via PCR, perhaps to determine if the breakpoint is recurrent across multiple samples. In this case, the breakpoint must be localized to an interval length that can be amplified via PCR, typically less than a few kilobases, and thus smaller clones are appropriate. On the other hand, in many cases rearrangement breakpoints are known to vary across kilobases in different patients [28]. Thus, approaches like Primer Approximation Multiplex PCR (PAMP) [28] that assay for variable genomic lesions in a patient population are useful, and the need for precise localization of breakpoints is reduced. Nevertheless, the success of PAMP relies on establishing reasonable boundaries of a rearrangement, so that appropriate primers tiling the region can be designed [29]. Our methodology provides these boundaries, and the combination of paired-end sequencing and PAMP is a powerful tool for identifying therapeutic targets and designing clinical diagnostics.

### Choice of Sequencing Parameters

There are several next-generation sequencing technologies now on the market, and others that soon will be commercially available. Information about the capabilities of many of these machines, particularly in regards to paired-end sequencing, is presently limited. In addition, the field is developing rapidly and any claims stated about read lengths, sequencing error rates, etc. are undergoing continual revision. While our analysis focused on several key parameters including number of paired reads, clone length, and percent of chimeric clones, in reality only some of these parameters are adjustable while others (e.g. error rate) are fixed by the chosen sequencing technology.

One issue not considered in our model that is closely tied to the sequencing technology is the mapping of reads to the reference genome. Different sequencing technologies produce reads of varying length and quality that can have a dramatic effect on the ability to map paired reads. On one extreme, conventional paired-end sequencing of cloned genomic fragments employed by current ESP studies [9],[21], yields high quality reads exceeding 500 bp. This enables the majority of reads outside of repeats and segmental duplications to be uniquely and accurately mapped to the reference genome. For example, with paired-end sequences 500 bp long, 11492 out of 19831 clones (58%) in the MCF7 study mapped uniquely [22], while with paired-end sequences 100 bp long 41% of paired reads using, mapped uniquely [15]. Newer sequencing technologies such as Illumina and ABI have even shorter reads (20 to 30 bp) and higher error rates [12],[13], and the ditag approach sequences only 18–20 base pairs from each end of the genomic fragment [11]. These shorter reads will be much more difficult to map, particularly when analyzing rearrangements. Moreover, unlike resequencing studies, where one can increase mapping efficiency using additional information that the end sequences are close together on the reference genome, detection of rearrangements specifically requires the accurate mapping of end sequences from distant locations on the genome. It would be informative to model the effect of different read accuracies and lengths on the ability to accurately resolve breakpoints.

### Organization of Cancer Genomes

Our simulations made certain simplifying assumptions about the character of cancer genomes. Most notably, we assumed that the size of the cancer genome (equal to the parameter *G* above) is known. Since many cancer genomes, particularly solid tumors, have extensive aneuploidy the actual size of a given genome might differ greatly from normal cells [30]. At the present time, it is difficult to calibrate the genome length parameter in our simulations, and pilot sequencing studies will be needed to assess the extent of amplification in these samples. Paired-end sequencing will naturally sample more from amplified regions. Although we did not explicitly simulate amplifications, it is clear that the probability of detecting amplified translocations is directly proportional to their relative amplification in the genome. Namely, as the number of copies, *a*, of a fusion point ζ increases, the probability of detection *P*_ζ_ increases, approximately following 1−*e* ^−*ca*^, assuming that the genome size is constant under the amplification. Since highly amplified regions can have complex organization due to duplication mechanisms [21],[31], many of the genome rearrangements detected in low coverage studies will likely be in these highly amplified and rearranged regions. Identification of non-amplified rearrangements might require extremely high coverage.

An additional consideration is whether cancer rearrangement breakpoints are biased to certain regions of the genome. For example, if rearrangement breakpoints are in highly repetitive regions, it might be difficult to map sequences that are too close to the breakpoints, and thus larger clones are appropriate. On the other hand, if there are multiple rearrangements clustered in a small genomic interval as observed in the multiple breakpoints found in some sequenced BACs and also in other recent sequencing studies [22],[32], larger clones would miss some of these rearrangements. Finally, genomic heterogeneity, particularly in primary tumor samples, reduces the effective coverage and thus the probability of detecting rearrangement breakpoints. Even a genomic lesion that is an important checkpoint in a cancer progression, might be difficult to detect in an admixed sample containing normal cells and cells from earlier developmental stages of the tumor. It is nearly impossible to determine how all of these factors will affect cancer sequencing strategies without further studies. Such pilot studies promise to provide a significant increase in new information about the extent of ploidy changes and heterogeneity.

### Extensions and Applications

Our formula for the probability of a fusion gene is readily extended to fusions of other genomic features. For example, we can compute the probability of regulatory fusions that result from the fusion of the promoter of one gene to the coding region of another gene. Other genomic assays such as array comparative genomic hybridization (CGH) can be used in combination with paired-end sequencing. Array CGH identifies breakpoints involved in deletions and amplifications at average resolutions of less than 10 kb [33],[34]. If this information overlaps paired-end sequencing data (such as the case with an amplified translocation like BCAS4/BCAS3) it might be possible to improve the resolution of the breakpoint interval defined by a paired-end sequencing approach. As next-generation technologies mature and the cost of sequencing declines, paired-end sequencing of cancer genomes will inevitably provide highly reliable and precise detection of fusion genes. Application of these technologies will permit the systematic analysis of all classes of genomic events that lead to cancer and will shed new light on the genetic and genomic basis of cancer.

## Methods

### Mapping and Clustering of End Sequences

We assume that each clone *C* is end-sequenced and the ends are mapped uniquely to the reference human genome sequence. Thus, each clone *C* corresponds to a pair (*x_C_*,*y_C_*) of locations in the human genome where the end sequences map. In addition, an end sequence may map to either DNA strand, and so each mapped end has a sign (+ or −) indicating the mapped strand. We call such a signed pair an end sequence pair (*ES pair*). In general the length (insert size) *L_C_* of the clone *C* is unknown, but is restricted to be in a range [*L_min_*,*L_max_*]. For most clones the observed distance between mapped ends will lie within this range and the ends will have opposite, convergent orientations: i.e. the corresponding ES pair will have the form (+*x*,−(*x*+*L_C_*)). Following [20] we call such ES pairs *valid pairs* because these indicate no rearrangement in the cancer genome. We use the distribution of distance |*y*|−|*x*| between the ends of valid pairs to define an empirical distribution of clone lengths (cf. Figure S1).

If a pair (*x_C_*,*y_C_*) has ends with non-convergent orientation or whose distance |*y*|−|*x*| is greater than *L_max_* or smaller than *L_min_*, we say that (*x_C_*,*y_C_*) is an *invalid pair*. The set of breakpoints (*a*,*b*) that are consistent with the invalid pair (*x_C_*,*y_C_*) is determined by the inequalities [35]

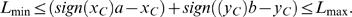
(6)Throughout the paper, we assume (without loss of generality) that *sign*(*x_C_*) = *sign*(*y_C_*) = + so that *a*≥*x_C_* and *b*≥*y_C_*.

### Validating Fusion Predictions by Sequencing

Clones containing predicted fusion genes were draft sequenced (1× coverage) by subcloning into 3 kb plasmids as described in [22]. Assembly of these sequences and alignment to the reference human genome identified either the precise fusion point, or identified a plasmid containing the fusion point thereby localizing the breakpoint to 3 kb.

### Computing Fusion Probability

Define *C*_(*a*,*b*)_ as the event that a clone *C* from the cancer genome with corresponding invalid pair (*x_C_*,*y_C_*) overlaps a breakpoint (*a*,*b*) of a reference genome. Assume w.l.o.g. that the invalid pair (*x_C_*,*y_C_*) is oriented such that *a*≥*x_C_* and *b*≥*y_C_*. The length *L_C_* of the clone is then equal to
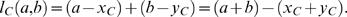
(7)Thus, the event *C*_(*a*,*b*)_ implies the event *L_C_* = *l_C_*(*a*,*b*), allowing us to express the probability of occurrence of breakpoint (*a*,*b*) in terms of the distribution on the lengths of clones. Let *N_C_*[*s*] denote the number of discrete breakpoints (*α*,*β*) such that *α*≥*x_C_*, *β*≥*y_C_*, and *α+β* = *s*. Then
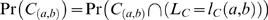
(8)

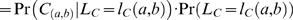
(9)

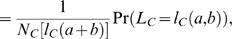
(10)where the last equality follows from Equation 7 and the assumption that all breakpoints are equally likely.

Now consider a pair of genes spanning genomic intervals *U* and *V*. An in-frame fusion transcript is possible if and only if *exactly* one of the genes is on the “+” strand and the other is on the “−” strand. In this case, the probability of a fusion gene being formed between these two genes given a clone *C* is the probability that the breakpoint (*a*,*b*) in *C* is also in *U*×*V*. This probability is
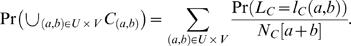
(11)Otherwise, if the genes are both on the same strand then an in-frame fusion transcript is impossible, and we define the fusion probability to equal zero. A similar analysis yields fusion gene probabilities in the cases of invalid pairs with other signs, by considering pairs of genes with compatible orientations. In the simple case, we assume that the clone lengths are uniformly distributed over the range [*L_min_*,*L_max_*], so that
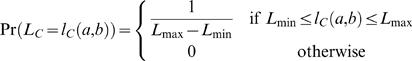



In this case, Equation 11 gives the fraction of the trapezoid (Equation 1) that intersects *U*×*V*. A more accurate distribution of clone lengths is obtained from the empirical distribution of distance between ends of valid ES pairs (Figure S1), and this distribution can also used to compute Pr(*L_C_* = *l_C_*(*a*,*b*)).

Next, we extend the equations to include the case when a set {*C* ^(1)^,*C* ^(2)^,…} of multiple clones overlap the breakpoint (*a*,*b*). Define *C* to be the event that all clones overlap the same breakpoint. Then
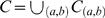
where
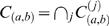
is the event that all clones *C* ^(*j*)^ overlap the breakpoint (*a*,*b*). Thus, the probability of (*a*,*b*) being the breakpoint given that all clones overlap it is given by
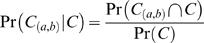
(12)

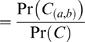
(13)

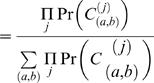
(14)Here, Equation 13 follows from the fact that *C*_(*a*,*b*)_ implies *C*, and Equation 14 follows from the independence of clones. This allows us to compute the probability that the genes spanning genomic intervals *U* and *V* fuse by
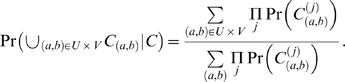
(15)


### Algorithms for Efficient Probability Computation

The naive approach for computing Pr(∪_(*a*,*b*)∈*U*×*V*_ *C*_(*a*,*b*)_|*C*) in Equation 15 is to compute 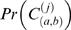 over all (*a*,*b*) and all clones *C* ^(*j*)^, which is time consuming. We exploit several features of this equation to make the computation more efficient. First, it is not necessary to compute 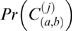 over all (*a*,*b*) in *U*×*V*, but only those (*a*,*b*) contained in the intersection of *all* of the trapezoids defined by the clones. Second, Equation 10 implies that
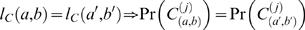
Finally, since *l_c_*(*a*,*b*) = (*a*+*b*)−(*x_C_*−*y_C_*) the points (*a*,*b*) with equal values of *l_C_*(*a*,*b*) lie on a line with slope −1 (an antidiagonal). This provides a methodology for rapidly computing the probability of fusion.

For an integer *s*, define the *diagonal D_s_* as the set of integral points (*a*,*b*) on the line *a*+*b* = *s* that are overlapped by all clones. Thus,
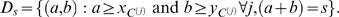
Hence, *D* = ∪*_s_D_s_* is the set of breakpoints that are overlapped by all clones. Define the *diagonal probability* as a product of the probabilities of these clone lengths
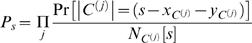
Then, we have
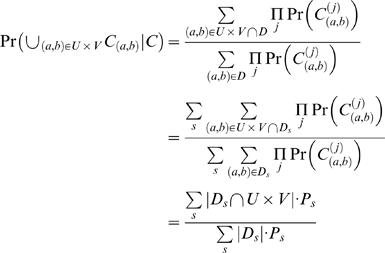
Thus we compute |*D_s_*|, |*D_s_*∩*U*×*V*|, *P_s_*, for all values of *s* intersected by all clones. This is more efficient than Equation 15, since there are relatively few diagonals with *P_s_*>0 and |*D_s_*|>0.

### Detection of Fusion Points

We now compute the probability of detecting a fusion point and the expected number of fusion points that are detected as a function of the number and length of clones that are end-sequenced. Recall that a *breakpoint* (*a*,*b*) is defined as a pair of non-adjacent coordinates *a* and *b* in the reference genome that are adjacent in the cancer genome, and a *fusion point* is defined as the coordinate ζ in the cancer genome such that *a* maps to ζ and *b* maps to ζ+1. Assume that *N* clones, each of length *L*, are end sequenced from a cancer genome of size *G*. We assume that the left endpoint of each clone is selected uniformly at random from the cancer genome. Then a fusion point ζ is detected if a clone contains it. Thus, the probability *P*_ζ_ of detection is given by [25],[26]

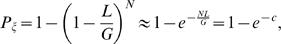
(16)where 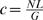 is the clonal coverage. Suppose there are *M* fusion points in the cancer genome, and define the random variables *X*_1_,…,*X_M_* by *X_i_* = 1 if the *i*-th fusion point is covered and *X_i_* = 0, otherwise. Then
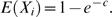
The expected number of fusion points detected is given by
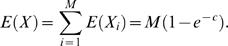
Using the Poisson approximation with *λ* = *M*(1−*e* ^−*c*^)
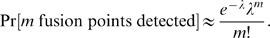
Given *m* observed fusion points, the maximum likelihood estimator *M̂* of the total number of fusion points is
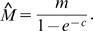
(17)


### Localization of Fusion Points

If one or more clones contain a fusion point ζ, the *localization* of ζ is defined as the length of the shortest interval that contains ζ according to the mapped locations of the clone ends. The localization is generally improved (i.e. decreased) when more clones contain a fusion point. Define Θ_ζ_ as the intersection of all clones that cover ζ (Figure 4). We compute the probability distribution on the length of Θ_ζ_ as follows. Following Lander-Waterman [26], we assume that the left endpoints of clones follow a Poisson process with intensity 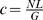 on the interval *G*. Θ_ζ_ is determined by the left endpoint of the right-most clone that contains ζ and the right endpoint of the left-most clone that contains ζ. Define for 0≤*j*≤*L*−1 as the event in which the right-most clone has its left endpoint *j* nucleotides to the left of ζ. Correspondingly, define *B_j_*, 1≤*j*≤*L* as the event that the left-most clone has its right endpoint *j* nucleotides to the right of ζ. The event *A_j_* occurs when there is a clone with left endpoint at ζ−*j* and no clones with left endpoints in the interval *j* nucleotides to the right of ζ−*j*, and similarly for *B_j_*. Therefore,
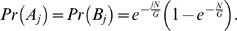
(18)The events are mutually exclusive for all *j*, and likewise for . Thus, we can express *P*_ζ_ as
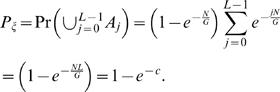
(19)Note that if *s*<*L*, then *A_s_*_−*j*_ and *B_j_* are independent for all *j*. To compute the probability distribution on |Θ_ζ_|, we have two cases. For *s*<*L*,
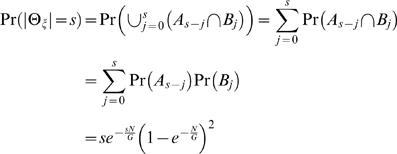
(20)The event |Θ_ζ_| = *L* requires all clones covering ζ to have the same left endpoint. Therefore
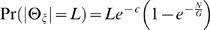
(21)We can compute the expected length of Θ_ζ_ conditioned on ζ being covered by a clone; otherwise Θ_ζ_ is undefined. Since the event |Θ_ζ_|≤*L* occurs only when ζ is covered, we have
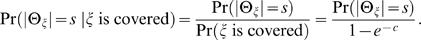
(22)Combining 20, 21, and 22 obtains
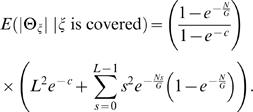
(23)We note that the sum in the above formula has a closed form solution:
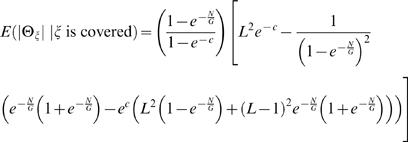
(24)


Because of the presence of chimeric clones, it is be useful to consider a fusion point ζ to be detected if is it covered by a *cluster* of 2 or more invalid pairs. In this case,
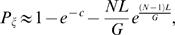
(25)and
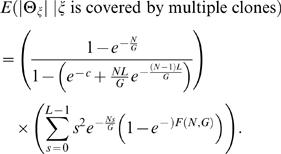
(26)


It is also useful to compute the probability that two or more chimeric clones form a cluster. Let *N* be the total number of paired reads as defined above and *q* be the probability that a mapped clone is chimeric. If we assume that the distribution of clone lengths has mean *L* and is uniformly distributed in the interval [*L*_min_,*L*_max_], then
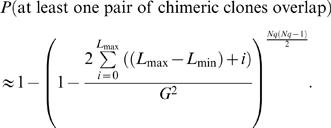
(27)


## Supporting Information

Text S1 Supporting Methods.
(0.05 MB PDF)

Figure S1 Distribution of MCF7 clone lengths. The mean for this distribution is 122 kb, and the standard deviation is 24 kb. Fusion Probabilities in Table 1 are computed using this distribution and the putative fusion regions for each gene pair (see Methods).
(0.03 MB PDF)

Figure S2 Length of a breakpoint region (BPR) for varying amounts of clonal coverage. The blue curve shows the expected length (Equation 5), while the red curve is the average observed length over 50 simulations.
(0.03 MB PDF)

Figure S3 Clone length vs. *P*_ζ_ vs. |Θ_ζ_| for varying *N*. A clear trade-off can be observed. Larger clone lengths yield higher *P*_ζ_ (detection probability), compared to smaller clone lengths, which have the advantage of better localization (smaller |Θ_ζ_|). Different lines originating from 0 refer to different number of reads. As the number of reads grows, the trade-off converges to high detection, and better localization. (A) shows values in a mesh graph, while (B) shows raw values.
(0.45 MB PDF)

Figure S4 The effect of clone length and number of paired reads on *P*_ζ_ and |Θ_ζ_|. (A) *P*_ζ_ increases as the number of paired reads *N* or clone length *L* increases, but is constant as a function of *N*/*L*. (B) |Θ_ζ_| decreases as the number of paired reads increases or the clones length decreases. Note that all axes are log values (with the exception of *P*_ζ_ in [A]).
(0.42 MB PDF)

Figure S5 *P*_ζ_ and |Θ_ζ_| for different *L* and *N*. (A) The probability of detecting a fusion point, *P*_ζ_, for different clone lengths and varying number of mapped paired reads. (B) The expected length of a breakpoint region, |Θ_ζ_|, around a fusion point (assuming that the fusion point is contained in a clone).
(0.18 MB PDF)

Figure S6 The number of paired-reads (and resulting *E*(|Θ_ζ_|)) needed to obtain a *P*_ζ_ of 0.99 for clone lengths varying from 1 to 150 kb. The x-axis indicates clone length, *L*, the y-axis indicates reads, *N*, and the alternate y-axis shows |Θ_ζ_|. The vertical line indicates the intersection point between the two lines at ∼16,000 bp.
(0.33 MB PDF)

Figure S7 Average fusion probability vs. number of mapped reads. The average fusion probability with mean and standard deviations as a function of *N*, the number of mapped paired reads. The x-axis represents the number of clones sequenced, *N*. The simulated fusion genes were 200 kb.
(0.06 MB PDF)

Figure S8 Effect of chimeric clones. The probability of observing at least one chimeric cluster for a fixed number of paired reads as a function of the percent of chimeric clones indicates that the observed rate of chimerism is lower for smaller clones. (A) 1 kb clones, (B) 10 kb clones, (C) 40 kb clones, and (D) 150 kb clones.
(0.05 MB PDF)
