# Supplementary material for: Evaluation of Paired-End Sequencing Strategies for Detection of Genome Rearrangements in Cancer
Source: PLoS Comput Biol. 2008 Apr 25;4(4):e1000051. doi: 10.1371/journal.pcbi.1000051 (PMC2278375; doi:10.1371/journal.pcbi.1000051)
Supplement: Text S1 — Supporting Methods. (0.05 MB PDF) [file pcbi.1000051.s001.pdf]

Simulations

Calculation of P_ζ and $E(|\Theta_\zeta)|$

In order to generate the observed distribution of $|\Theta_\zeta|$ a random position, ζ, was chosen in an interval of size G, which represents the tumor genome. Reads were simulated by selected random numbers in the range $[0, G-L)$, where L corresponds the size of the clone (150kb unless otherwise noted). In each iteration $N = cG/L$ clones are generated. If a random number is observed at positions $[\zeta - L, \zeta)$ then we compute the observed length of Θ_ζ by ordering the set of clones overlapping ζ and selecting the rightmost clone to define the start of interval and the leftmost point to define the end of the interval. The average of all iterations for which a clone is observed to *span* the breakpoint is computed for each c.

Calculation of Fusion Probabilities for Artificial Fusion Genes

In order to simulate fusion events, in each graph, a fusion gene was created by randomly selecting gene lengths from the distribution of genes in the genome (derived from the "known genes" table at the UCSC Genome browser. and randomly fusing them to create the desired fusion gene length. Random paired-reads were then generated on these rearranged genomes (of length normally distributed around mean L). For *each* artificial fused gene 100 such simulations were performed at *each* clone size- 100 artificial fusion genes were created for each read value and clone size (corresponding to 10000 simulations at each datapoint). All invalid reads were grouped into clusters. This set of invalid pairs, along with the the complete list of genomic positions for all known genes *as well as* the two artificial genes, was fed as input to our algorithm. Note, that if no paired read spanning the fusion gene was observed at a given datapoint, then a 0 was returned as the fusion probability for that iteration.

Sensitivity and Selectivity under Random Rearrangements

As in the previous section we began with a diploid reference genome. 100 random rearrangement events (of average size 1Mb) were performed on the reference genome. Subsequently paired reads were generated on the rearranged genome, with 1% of paired reads being chimeric. This rearranged genome was analyzed explicitly, by identification of each breakpoint (a, b), to determine all "true" fusion genes. For each simulated paired-read set the complete set of fusion probabilities for all invalid clusters (including singletons) was generated. The predicted fusion genes were partitioned into "True Positives" and "False Positives" in each set. Counts corresponding to the number of "true" and "false" fusion genes above specific fusion probabilities ($> 0, > 0.05, > 0.1... > .95$) were determined for each simulation. The average of such counts over 50 simulations (for a given clone size and number of paired reads) is plotted in Figure 8.
