# Supplementary material for: Evaluation of Paired-End Sequencing Strategies for Detection of Genome Rearrangements in Cancer
Source: PLoS Comput Biol. 2008 Apr 25;4(4):e1000051. doi: 10.1371/journal.pcbi.1000051 (PMC2278375; doi:10.1371/journal.pcbi.1000051)
Supplement: Figure S1 — Distribution of MCF7 clone lengths. The mean for this distribution is 122 kb, and the standard deviation is 24 kb. Fusion Probabilities in Table 1 are computed using this distribution and the putative fusion regions for each gene pair (see Methods). (0.03 MB PDF) [file pcbi.1000051.s002.pdf]

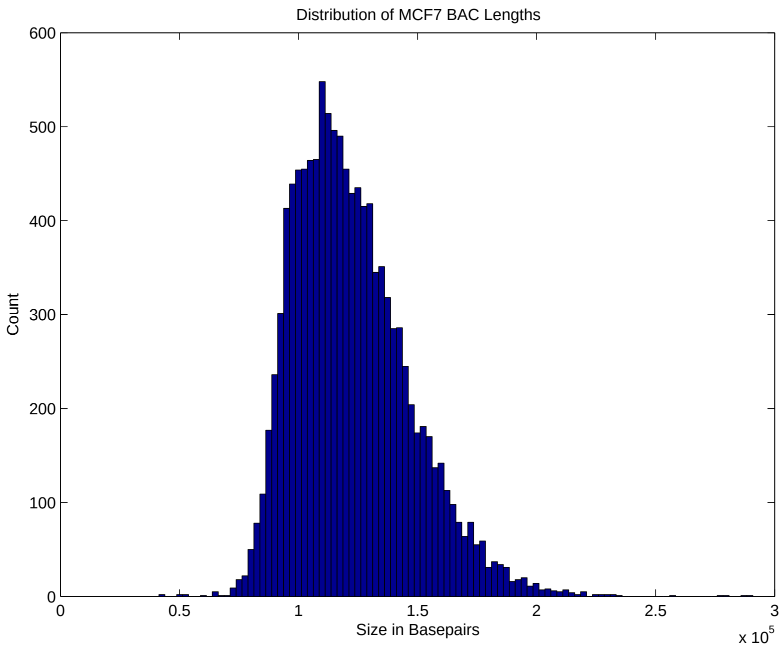

Figure 1: **Distribution of MCF7 clone lengths.** The mean for this distribution is 122 kb, and the standard deviation is 24 kb. Fusion Probabilities in Table 1 are computed using this distribution and the putative fusion regions for each gene pair (see **Methods**).
