# Supplementary material for: Evaluation of Paired-End Sequencing Strategies for Detection of Genome Rearrangements in Cancer
Source: PLoS Comput Biol. 2008 Apr 25;4(4):e1000051. doi: 10.1371/journal.pcbi.1000051 (PMC2278375; doi:10.1371/journal.pcbi.1000051)
Supplement: Figure S2 — Length of a breakpoint region (BPR) for varying amounts of clonal coverage. The blue curve shows the expected length (Equation 5), while the red curve is the average observed length over 50 simulations. (0.03 MB PDF) [file pcbi.1000051.s003.pdf]

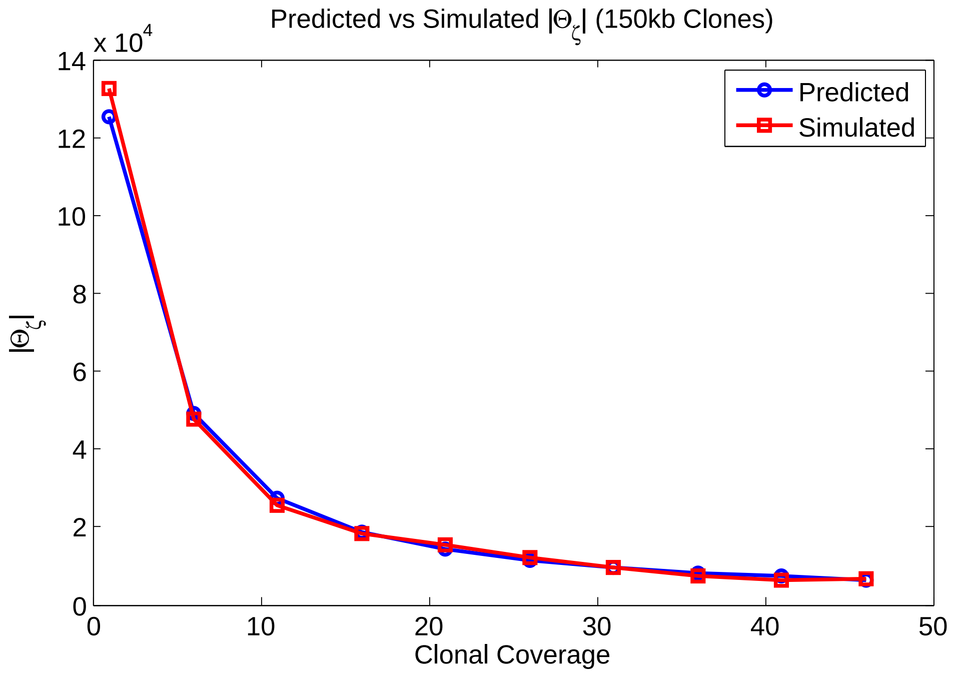

Figure 2: **Length of a breakpoint region (BPR) for varying amounts of clonal coverage.** The blue curve shows the expected length (Equation 5), while the red curve is the average observed length over 50 simulations.
