# Supplementary material for: Evaluation of Paired-End Sequencing Strategies for Detection of Genome Rearrangements in Cancer
Source: PLoS Comput Biol. 2008 Apr 25;4(4):e1000051. doi: 10.1371/journal.pcbi.1000051 (PMC2278375; doi:10.1371/journal.pcbi.1000051)
Supplement: Figure S3 — Clone length vs. P ζ vs. |Θζ| for varying N. A clear trade-off can be observed. Larger clone lengths yield higher P ζ (detection probability), compared to smaller clone lengths, which have the advantage of better localization (smaller |Θζ|). Different lines originating from 0 refer to different number of reads. As the number of reads grows, the trade-off converges to high detection, and better localization. (A) shows values in a mesh graph, while (B) shows raw values. (0.45 MB PDF) [file pcbi.1000051.s004.pdf]

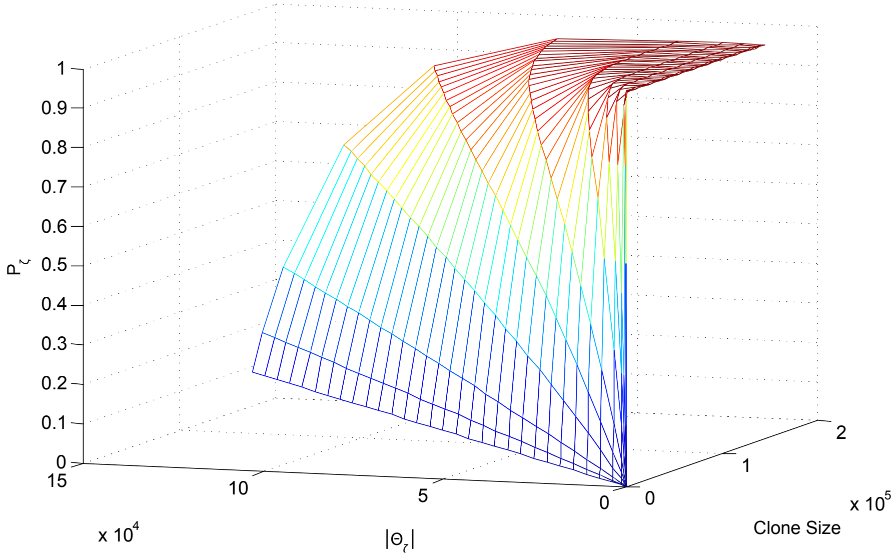

(a)

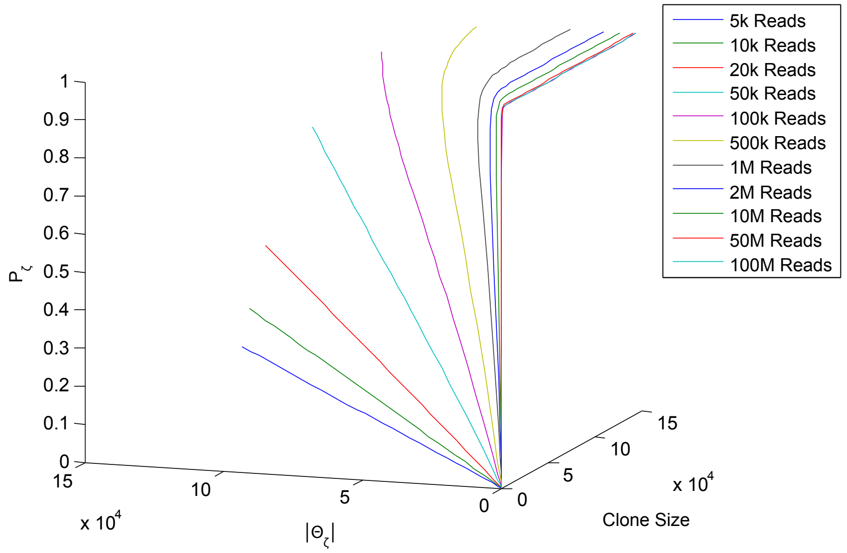

(b)

Figure 3: **Clone length vs.** P_ζ **vs.** $|\Theta_\zeta|$ **for varying** N**.** A clear trade-off can be observed. Larger clone lengths yield higher P_ζ (detection probability), compared to smaller clone lengths, which have the advantage of better localization (smaller $|\Theta_\zeta|$). Different lines originating from 0 refer to different number of reads. As the number of reads grows, the trade-off converges to high detection, and better localization. (a) shows values in a mesh graph, while (b) shows raw values.
