# Supplementary material for: Evaluation of Paired-End Sequencing Strategies for Detection of Genome Rearrangements in Cancer
Source: PLoS Comput Biol. 2008 Apr 25;4(4):e1000051. doi: 10.1371/journal.pcbi.1000051 (PMC2278375; doi:10.1371/journal.pcbi.1000051)
Supplement: Figure S4 — The effect of clone length and number of paired reads on P ζ and |Θζ|. (A) P ζ increases as the number of paired reads N or clone length L increases, but is constant as a function of N/L. (B) |Θζ| decreases as the number of paired reads increases or the clones length decreases. Note that all axes are log values (with the exception of P ζ in [A]). (0.42 MB PDF) [file pcbi.1000051.s005.pdf]

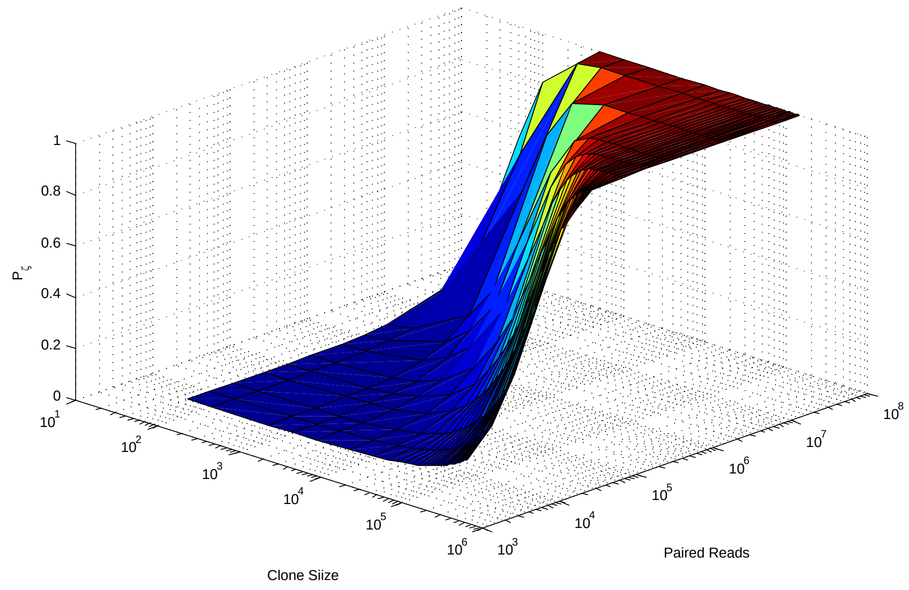

(a)

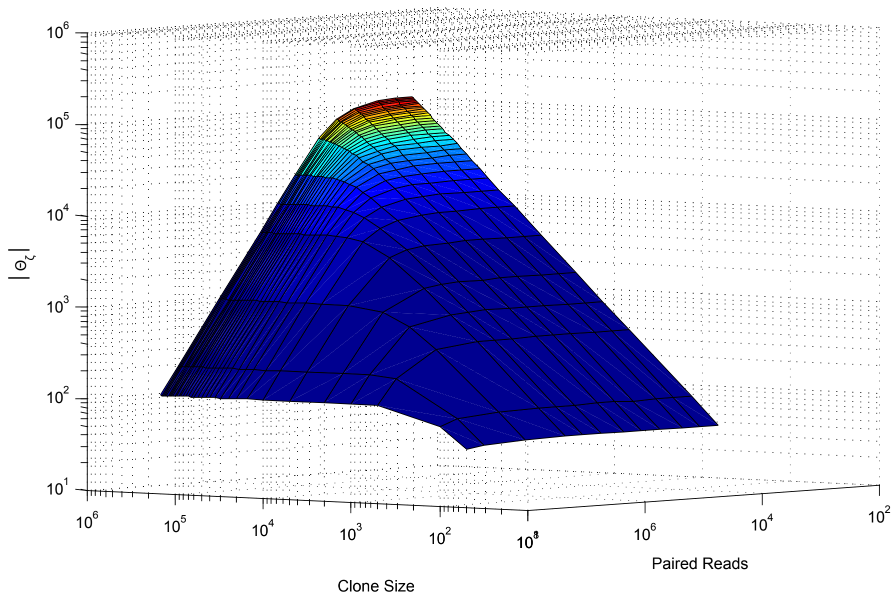

(b)

Figure 4: **The effect of clone length and number of paired reads on P_ζ and $|\Theta_\zeta|$.** (a) P_ζ increases as the number of paired reads N or clone length L increases, but is constant as a function of N/L. (b) $|\Theta_\zeta|$ decreases as the number of paired reads increases or the clones length decreases. Note that all axes are log values (with the exception of P_ζ in (a)).
