# Supplementary material for: Evaluation of Paired-End Sequencing Strategies for Detection of Genome Rearrangements in Cancer
Source: PLoS Comput Biol. 2008 Apr 25;4(4):e1000051. doi: 10.1371/journal.pcbi.1000051 (PMC2278375; doi:10.1371/journal.pcbi.1000051)
Supplement: Figure S5 — P ζ and |Θζ| for different L and N. (A) The probability of detecting a fusion point, P ζ, for different clone lengths and varying number of mapped paired reads. (B) The expected length of a breakpoint region, |Θζ|, around a fusion point (assuming that the fusion point is contained in a clone). (0.18 MB PDF) [file pcbi.1000051.s006.pdf]

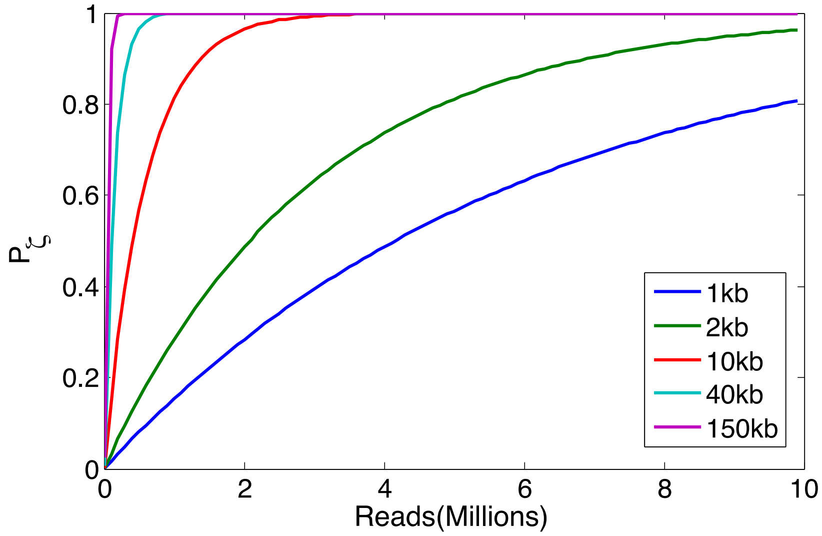

(a)

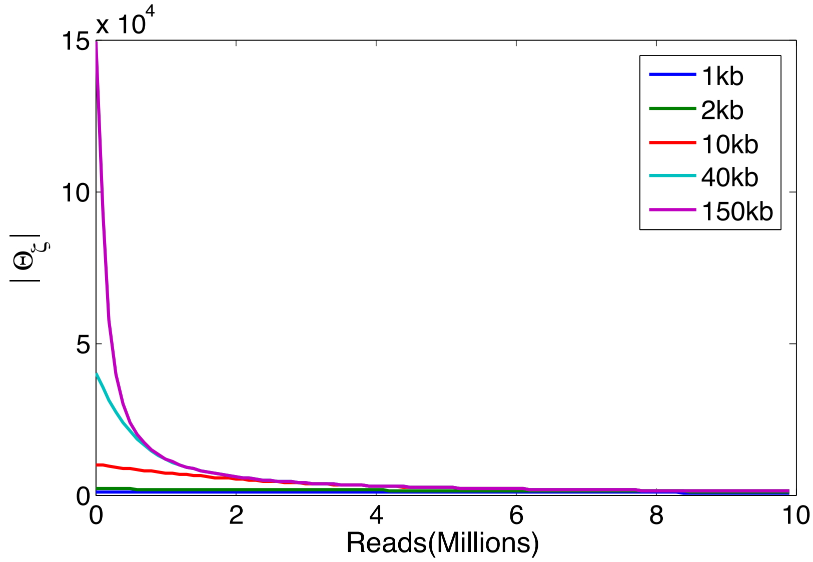

(b)

Figure 5: P_ζ **and** $|\Theta_\zeta|$ **for different** L **and** N**.** (a) The probability of detecting a fusion point, P_ζ, for different clone lengths and varying number of mapped paired reads. (b) The expected length of a breakpoint region, $|\Theta_\zeta|$, around a fusion point (assuming that the fusion point is contained in a clone).
