# Supplementary material for: Evaluation of Paired-End Sequencing Strategies for Detection of Genome Rearrangements in Cancer
Source: PLoS Comput Biol. 2008 Apr 25;4(4):e1000051. doi: 10.1371/journal.pcbi.1000051 (PMC2278375; doi:10.1371/journal.pcbi.1000051)
Supplement: Figure S6 — The number of paired-reads (and resulting E(|Θζ|)) needed to obtain a P ζ of 0.99 for clone lengths varying from 1 to 150 kb. The x-axis indicates clone length, L, the y-axis indicates reads, N, and the alternate y-axis shows |Θζ|. The vertical line indicates the intersection point between the two lines at ∼16,000 bp. (0.33 MB PDF) [file pcbi.1000051.s007.pdf]

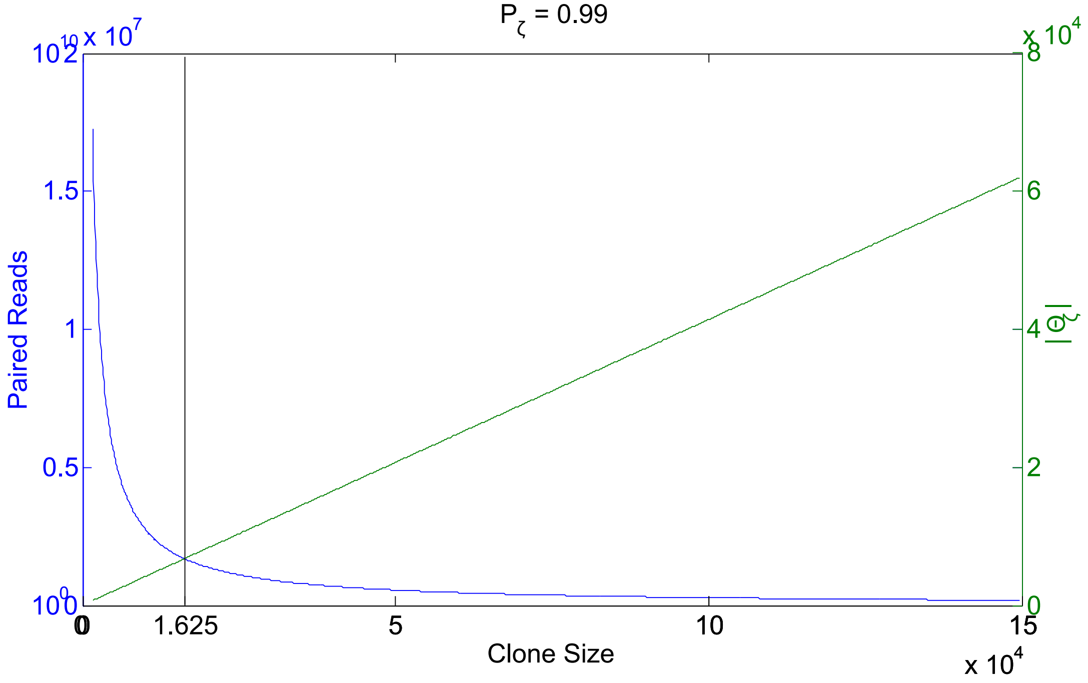

Figure 6: **The number of paired-reads (and resulting $E(|\Theta_\zeta|)$) needed to obtain a P_ζ of 0.99 for clone lengths varying from 1 to 150kb.** The x-axis indicates clone length, L, the y-axis indicates reads, N, and the alternate y-axis shows $|\Theta_\zeta|$. The vertical line indicates the intersection point between the two lines at $\sim$ 16000bp.
