# Supplementary material for: Evaluation of Paired-End Sequencing Strategies for Detection of Genome Rearrangements in Cancer
Source: PLoS Comput Biol. 2008 Apr 25;4(4):e1000051. doi: 10.1371/journal.pcbi.1000051 (PMC2278375; doi:10.1371/journal.pcbi.1000051)
Supplement: Figure S7 — Average fusion probability vs. number of mapped reads. The average fusion probability with mean and standard deviations as a function of N, the number of mapped paired reads. The x-axis represents the number of clones sequenced, N. The simulated fusion genes were 200 kb. (0.06 MB PDF) [file pcbi.1000051.s008.pdf]

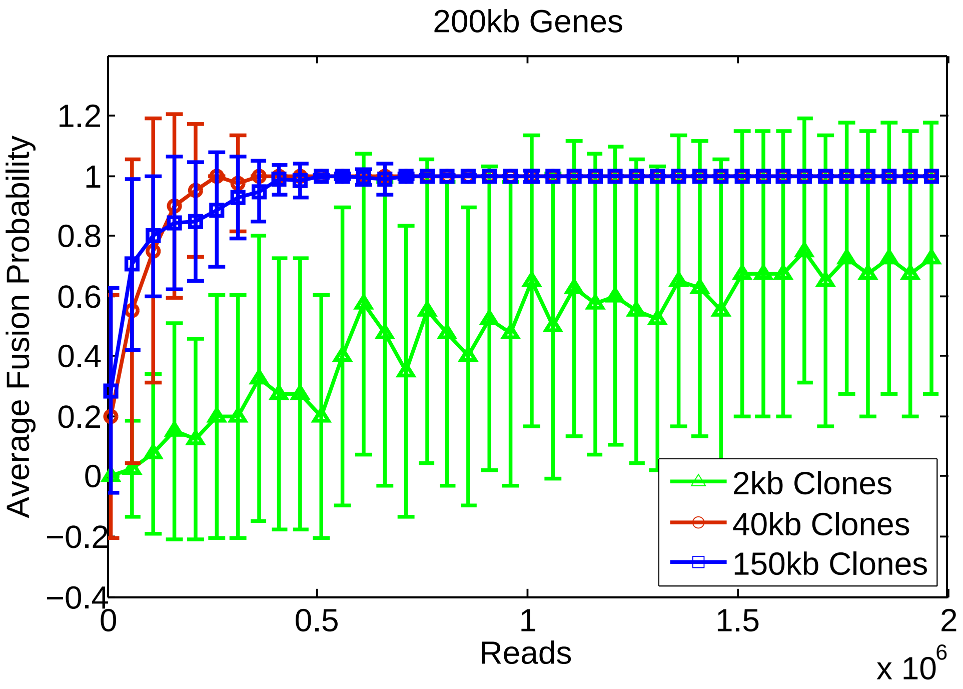

Figure 7: **Average fusion probability vs. number of mapped reads.** The average fusion probability with mean and standard deviations as a function of N, the number of mapped paired reads. The x-axis represents the number of clones sequenced, N. The simulated fusion genes were 200kb.
