# Supplementary material for: Evaluation of Paired-End Sequencing Strategies for Detection of Genome Rearrangements in Cancer
Source: PLoS Comput Biol. 2008 Apr 25;4(4):e1000051. doi: 10.1371/journal.pcbi.1000051 (PMC2278375; doi:10.1371/journal.pcbi.1000051)
Supplement: Figure S8 — Effect of chimeric clones. The probability of observing at least one chimeric cluster for a fixed number of paired reads as a function of the percent of chimeric clones indicates that the observed rate of chimerism is lower for smaller clones. (A) 1 kb clones, (B) 10 kb clones, (C) 40 kb clones, and (D) 150 kb clones. (0.05 MB PDF) [file pcbi.1000051.s009.pdf]

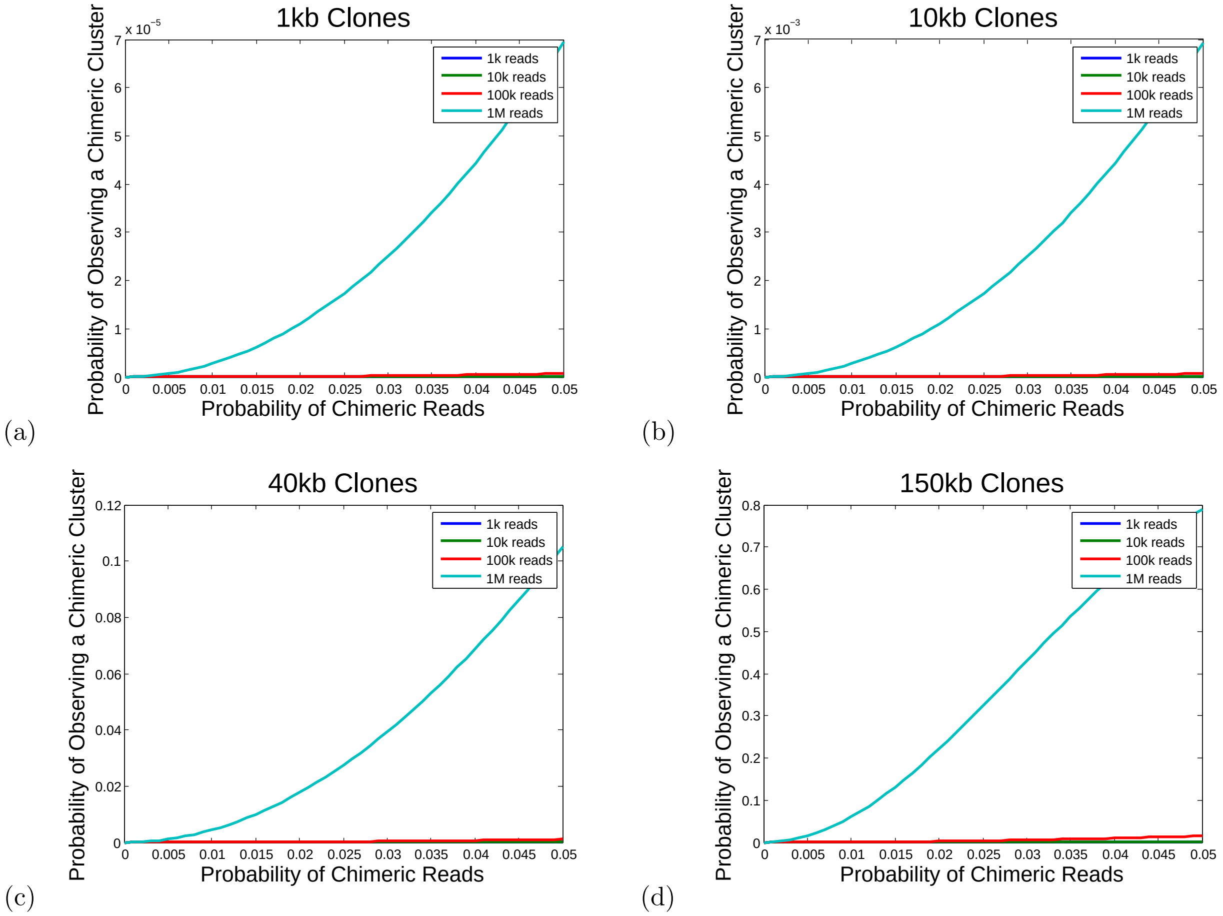

Figure 8: **Effect of chimeric clones**. Probability of observing at least one chimeric cluster vs. the percent of chimeric clones under equal number of paired reads indicates lower chimerism for smaller clones. (a) 1kb clones, (b) 10kb clones, (c) 40kb clones, and (d) 150kb clones.
